# Effects of Climatic Factors and Ecosystem Responses on the Inter-Annual Variability of Evapotranspiration in a Coniferous Plantation in Subtropical China

**DOI:** 10.1371/journal.pone.0085593

**Published:** 2014-01-22

**Authors:** Mingjie Xu, Xuefa Wen, Huimin Wang, Wenjiang Zhang, Xiaoqin Dai, Jie Song, Yidong Wang, Xiaoli Fu, Yunfen Liu, Xiaomin Sun, Guirui Yu

**Affiliations:** 1 Qianyanzhou Ecological Station, Key Laboratory of Ecosystem Network Observation and Modeling, Institute of Geographic Sciences and Natural Resources Research, Chinese Academy of Sciences, Beijing, China; 2 University of Chinese Academy of Sciences, Beijing, China; 3 State Key Laboratory of Hydraulics and Mountain River Engineering, Sichuan University, Chengdu, China; 4 Department of Geography, Northern Illinois University, DeKalb, Illinois, United States of America; 5 Tianjin Key Laboratory of Water Resources and Environment, Tianjin Normal University, Tianjin, China; Tennessee State University

## Abstract

Because evapotranspiration (ET) is the second largest component of the water cycle and a critical process in terrestrial ecosystems, understanding the inter-annual variability of ET is important in the context of global climate change. Eight years of continuous eddy covariance measurements (2003–2010) in a subtropical coniferous plantation were used to investigate the impacts of climatic factors and ecosystem responses on the inter-annual variability of ET. The mean and standard deviation of annual ET for 2003–2010 were 786.9 and 103.4 mm (with a coefficient of variation of 13.1%), respectively. The inter-annual variability of ET was largely created in three periods: March, May–June, and October, which are the transition periods between seasons. A set of look-up table approaches were used to separate the sources of inter-annual variability of ET. The annual ETs were calculated by assuming that (a) both the climate and ecosystem responses among years are variable (V_cli-eco_), (b) the climate is variable but the ecosystem responses are constant (V_cli_), and (c) the climate is constant but ecosystem responses are variable (V_eco_). The ETs that were calculated under the above assumptions suggested that the inter-annual variability of ET was dominated by ecosystem responses and that there was a negative interaction between the effects of climate and ecosystem responses. These results suggested that for long-term predictions of water and energy balance in global climate change projections, the ecosystem responses must be taken into account to better constrain the uncertainties associated with estimation.

## Introduction

Evapotranspiration (ET) is the second largest component of the water cycle, consuming a large proportion (60–90%) of annual precipitation [1]–[3]. ET plays an important role in terms of the energy and nutrient exchange in ecosystems [4]–[6] and affects many other important ecological processes [7]. Forests cover approximately 30% of the global land surface, but their evapotranspiration accounts for more than 45% of the global ET across terrestrial ecosystems [8]. In the context of climate change, forests are considered to provide important climate forcings and feedbacks [9]. While climate change may adversely affect ecosystem functions, forests could be managed to mitigate climate change [10]. As ET is an integral part of forest biogeophysical processes [9], meteorologists and ecologists require an understanding of how the forest ET responds to environmental drivers so that they can better predict how the biosphere will affect (or will be affected by) climate change [11]–[16].

The factors that control ET have been well documented for many ecosystems on intra-annual scales [17]–[20]. Evapotranspiration is controlled by abiotic and biotic factors, many of which are projected to change on multiple spatial and temporal scales [21]. Generally, ET is positively related to net radiation (R_n_), air temperature (T_a_), and the vapor pressure deficit (VPD) [12], [22]–[24]. However, soil water content (SW) sometimes becomes the main control factor under drought conditions [25]. As for biotic factors, leaf area index (LAI) [26] and additional model parameters that have been used to represent the canopy characteristics have influences on ET. For example, canopy conductance, which can be computed by inverting canopy resistance from the Penman-Monteith equation [27]–[30], can help estimate ET. These parameters were found to be closely related to ET, especially under drought stress [19], [31].

In contrast to intra-annual variability, the inter-annual variability of ET and its driving mechanisms are poorly understood. Although many forest evaporation datasets have been collected worldwide using the eddy covariance technique and compiled over years to decades [32], [33], only a small fraction of studies have published their long-term ET records. Furthermore, most of these studies have concentrated on tropical forests [20], [34], [35], and boreal forests [36], [37]. There have also been a few studies reporting the inter-annual variability of ET based on 3–5 year measurements in temperate forests [31], [38], [39]. Some of these studies have determined that the inter-annual variability of ET was small (coefficient of variation (CV) <10%) in contrast to the considerable year-to-year variation in annual rainfall [35], [37], [39]. Others who held the opposite views have insisted that the inter-annual variability of ET was significant and could not be neglected [38]. In addition, several models have been developed to study or predict long-term variation in ET [28]. However, these models may be inadequate for evaluating the inter-annual variability of ET because of their dependency on climatic factors [40]–[42].

The inter-annual variability of ET can be affected by biotic and abiotic factors, similar to those that operate on shorter time scales [21], [31]. In addition to the factors mentioned above, the leaf phenology should be given careful consideration. As noted by Xue et al. [38], the reason that Ohta et al. [37] could not fully explain the inter-annual variability of ET in a Siberian larch forest was because they neglected the inter-annual variability in the dates of leaf expansion. Zha et al. [36] determined that the inter-annual variability of ET was controlled by early spring soil temperatures, which are a key factor affecting the growing season length (GSL).

In this study, the sources of the inter-annual variability of ET were separated into two parts: (1) those induced by climatic factors (extreme climatic events included) and (2) those produced by ecosystem responses including the changes in phenology (e.g., GSL), ecosystem structure (e.g., LAI), and physiological changes in plants (e.g., stomatal and canopy conductance) [43], [44]. Some studies have proposed that ecosystem responses overwhelm the direct influences of environmental factors on the inter-annual variability of ET [5], [21], [34], but few researchers have separated the sources of inter-annual variability of ET into ‘biological’ and ‘climatological’ components because they are quite difficult to tease apart. However, in recent studies, the inter-annual variations of carbon fluxes were successfully separated into those driven by environmental factors or by biological factors with a novel statistical method [44], [46]. These studies suggested that it would be possible to separate the sources of inter-annual variability of ET because water processes are highly correlated with carbon assimilation in an ecosystem.

Southern China is characterized by a humid monsoon climate and has large subtropical coniferous plantations, which account for 41% of the total subtropical forest area [47]. Thus, it is important to conduct research on the carbon and water cycles in this region. Previous studies have indicated that the carbon fluxes are characterized by strong inter-annual variations due to seasonal droughts in the summer or low temperatures in the early spring [43], [48]. Considering that water and carbon cycles are directly coupled within the stomata through transpiration, photosynthesis, and respiration [12], evapotranspiration may respond to climatic drivers as do the carbon fluxes. Therefore, the goal of this study is to investigate whether the inter-annual variability of ET in this region is strong and to determine the main source of the inter-annual variability of ET.

Eight years (2003–2010) of continuous eddy-covariance measurements that were recorded at a subtropical coniferous forest site were analyzed. Based on previous research on the carbon cycle [44], a set of look-up tables (LUTs) were employed to separate the sources of the inter-annual variability of ET. The objectives of this study were to (1) characterize the seasonal and inter-annual variation of ET and (2) evaluate the contributions of the two types of driving forces, climatic factors and ecosystem responses, to the inter-annual variability of ET.

## Materials and Methods

### Site description

This study was conducted in a subtropical coniferous plantation (26°44′29″N, 115°03′29″E, 102 m in elevation above sea level) at Qianyanzhou Ecological Research Station (QYZ), which is owned by the Institute of Geographic Sciences and Natural Resources Research of the Chinese Academy of Sciences. No specific permits were required for the described field studies in the research site. The field studies did not involve endangered or protected species. The site is located in a typical red soil hilly region in south China, with a subtropical monsoon climate. The prevailing wind direction of this climate regime is north-northwest in the winter and south-southeast in the summer. The coniferous trees were planted around 1985. The dominated species are Masson pine (*Pinus massoniana* Lamb.), Slash pine (*Pinus elliottii* Englem.) and Chinese fir (*Cunninghamia lanceolata* Hook.), with a tree density of about 1460 stems ha^−1^ and total biomass of 106 t ha^−1^. The red soil is weathered from red sand rock, and soil texture is divided into 2.0–0.05 mm (17%), 0.05–0.002 mm (68%) and <0.002 mm (15%). Further details of QYZ site can be referred to Wen et al. [49] and Wang et al. [50].

**Figure 1:**
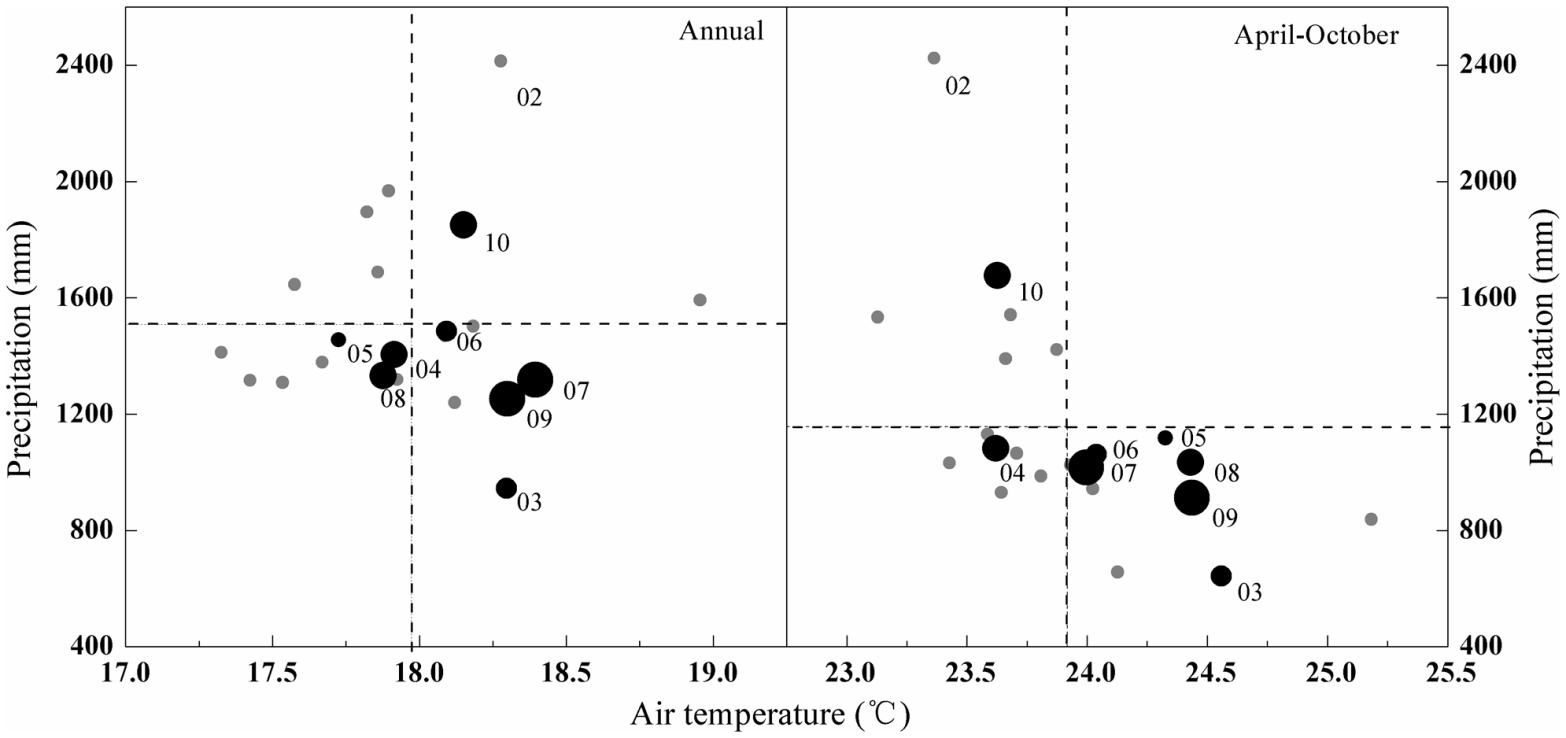
Cumulative precipitation versus average air temperature of the whole year and the growing season (April–October) during the period 1989–2010. For the period of 2003–2010, the size of black dot is approximately proportional to the ET amount. The two-digit numbers (YY) denote the years from 2003 to 2010 (20YY). Dotted lines represent the averages over the 20 year period.

According to meteorological observations from 1989–2010 at QYZ, the mean annual temperature is 18.0°C and mean annual precipitation is 1509.0 mm, while the growing season (April–October) mean values are 23.9°C and 1050.8 mm, respectively (Fig. 1). Eight years (2003–2010) of flux data were observed with the eddy covariance method in a subtropical forest plantation at QYZ and used for this study. During the eight observation years, the dataset represented much of the typical inter-annual variability at this site, including both typical and extreme climatic events. In 2002–2003, a typical El Niño event occurred, which resulted in an extreme summer drought in 2003 [51], and in 2009–2010, there was a strong La Niña event [52].

### Observation and instrumentation

**Figure 2:**
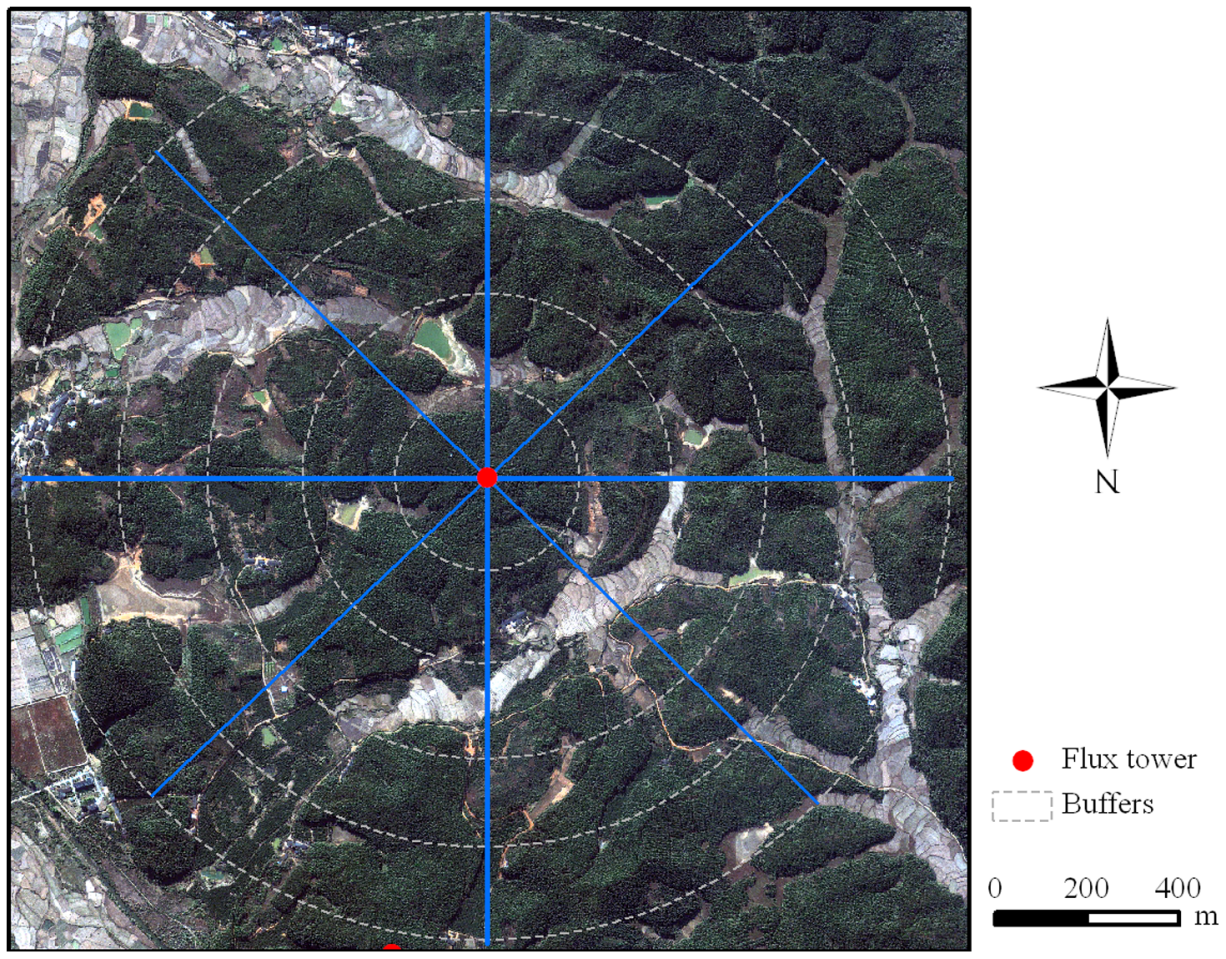
The IKONOS image (Nov. 7, 2003) around the QYZ flux tower (red point). The interval between buffer circles is 200[43].

The eddy covariance flux observation system was established in a subtropical coniferous plantation at QYZ site in 2002 (Fig. 2). The above-canopy flux was measured at a height of 39.6 m by instruments loaded on a ventilated tower. The wind velocity was detected by a 3-D sonic anemometer (Model CSAT3, Campbell Scientific Inc., USA), and the variations in CO_2_ and water vapor concentration were measured with a LI-7500 open-path CO_2_/H_2_O analyzer (Licor Inc., USA). All signals were sampled at a frequency of 10 Hz and the CO_2_ and H_2_O fluxes were calculated and recorded at 30 min intervals by a CR5000 datalogger (Campbell Scientific Inc., USA).

Air temperature and relative humidity sensors (Model HMP45C, Campbell) were mounted in ventilated shield at the heights of 23.6 m and 39.6 m above the ground. Soil temperature and soil water content were measured at the depths of 5 cm with thermocouples (105T and 107-L, Campbell), and TDR probes (Model CS615-L, Campbell), respectively. Radiation measurements were made using a four-component net radiometer (Model CNR-1, Kipp & Zonen, Netherlands), a pyranometer (Model CM11, Kipp & Zonen) and a quantum sensor of photosynthetically active radiation (Model LI190SB, Licor Inc.). Rainfall was monitored with a rain gauge (Model 52203, RM Young Inc., USA). Meteorological variables were sampled at 1 Hz, and 30 min average data were recorded with three CR10X dataloggers, and a CR23X datalogger with a 25-channel solid-state multiplexer (Campbell Scientific Inc., USA).

### Flux correction and gap filling

This study adopted the methods of calculating and correcting the carbon dioxide and water vapor fluxes that were presented by Wen et al. [48]. The CO_2_ and H_2_O fluxes were calculated every 30 minutes from the 10 Hz raw data. Processing of the flux data was performed using routine methods, including three-dimensional rotation [53], a correction for the effects of the fluctuations of air density (the Webb, Pearman and Leuning density correction (WPL correction)) [54], storage calculations and spurious data removal [43], [49], [55]. Spurious data caused by rainfall, water condensation or system failure were removed from the dataset. To avoid the possible underestimation of the fluxes under stable conditions at night, nighttime data (solar elevation angle<0) were excluded when the value of friction velocity (u_*_) was below the relevant thresholds, which were identified with the method described by Reichstein et al. [56]. The threshold values of u_*_ ranged from 0.16 to 0.22 m s^−1^, with an average value of 0.20 m s^−1^ for the years from 2003 to 2010. Approximately 23.8% of the half-hour data were excluded for the daytime and 85.4% were excluded for the nighttime. The data gaps were filled using the look-up table method [56]. Any data gaps in meteorological variables were filled using the mean diurnal variation method [57]. Further details of data processing are presented in the previous studies of ChinaFLUX [43], [48], [55], [58]. In this study, the H_2_O flux was considered to be ET of the whole ecosystem, as in previous studies [58], [59].

### Partitioning the sources of inter-annual variation in ET

To separate the effects of climate drivers and ecosystem responses on the inter-annual variability of carbon fluxes, Marcolla et al. [44] established a set of look-up tables (LUTs) according to selected key factors that influence the ecosystem carbon assimilation, such as temperature (T_a_) and photosynthetically active radiation (PAR). Using these LUTs, these researchers statistically separated the two sources of inter-annual variability in carbon fluxes. In this study, a similar methodology was applied, but the LUTs for ET were built with the environmental factors R_n_ and VPD, which have a strong influence on the ET and widely used in combination with Penman-Monteith Equation [12].

To create the LUTs, the half-hour flux values and corresponding meteorological data were integrated to daily scale for each year. Then, the data were classified according to R_n_ (class width 1 Wm^−2^) and VPD (class width 0.1 kPa). With the LUTs, we could then use the meteorological data (R_n_ and VPD) to estimate a corresponding ET value. Specifically, the ETs were calculated in three different ways:

1. The yearly LUTs were established using the meteorological data (R_n_ and VPD) and the corresponding ET. Then, the yearly observed meteorological data from the entire time series (DOY (day-of-year) 1–365 (366 for leap year)) was used to reproduce the ET series according to the yearly LUTs. The ET series and the inter-annual variability of ET calculated with this method can be considered to include effects of both climatic factors and the ecosystem responses (V_cli-eco_).
2. To isolate the climate factors, an average table that included meteorological data and ET for the entire time series from DOY 1 to 366 (including leap years) was established based on the eight-year data. Then, the LUT_avg_ based on the average table was established. In the LUT_avg_, the ETs represent the average ecosystem responses for the eight years that were measured. Finally, the LUT_avg_ is looked up using the yearly observed meteorological data from the entire time series to reassign ET series. The inter-annual variability of ET that is calculated through this method can be considered to be the inter-annual variability caused by variable climatic factors (V_cli_).
3. To isolate the ecosystem responses, the eight-year averaged daily meteorological data with entire time series (DOY 1–366) can be used to look up the yearly LUTs established in approach (1) to reproduce ET series for each year. The inter-annual variability of ET calculated through this method can be considered to be the inter-annual variability caused by variable ecosystem responses (V_eco_).

The standard deviations of the yearly values of ET that were obtained through the three approaches (V_cli-eco_, V_cli_ and V_eco_) were used as estimates of what portion of the inter-annual variability of ET was generated from which sources. Then the interaction of climate drivers and ecosystem responses was analyzed based on these results.

### Correlation between climatic drivers and ecosystem evapotranspiration

To identify the relationship between climatic drivers and ET, we selected five climatic factors, net radiation (R_n_), air temperature (T_a_), precipitation (P), vapor pressure deficit (VPD) and soil water content (SW), that were important according to the previous studies [31], [36], [37], [60]. We analyzed their correlations with the ecosystem ET on the annual scale, placing emphasis on the time lag effects between the climatic factors and ET.

For this purpose, annual average T_a_, SW, VPD and cumulative R_n_, precipitation and ET were calculated. Multiple “yearly” statistics (approximately 80 values from the eight years) were obtained using the 12-month intervals and shifting them one month at a time [44], [45]. To investigate the lag of the ET's response to the climatic factors, we shifted the climatic series backward one month at a time (up to twelve months) to calculate the correlations between climate drivers and ET. Student's *t*–tests were applied to verify the statistical significance of correlation coefficients.

## Results

### Seasonal and inter-annual variation of ecosystem evapotranspiration and the corresponding environmental factors

**Figure 3:**
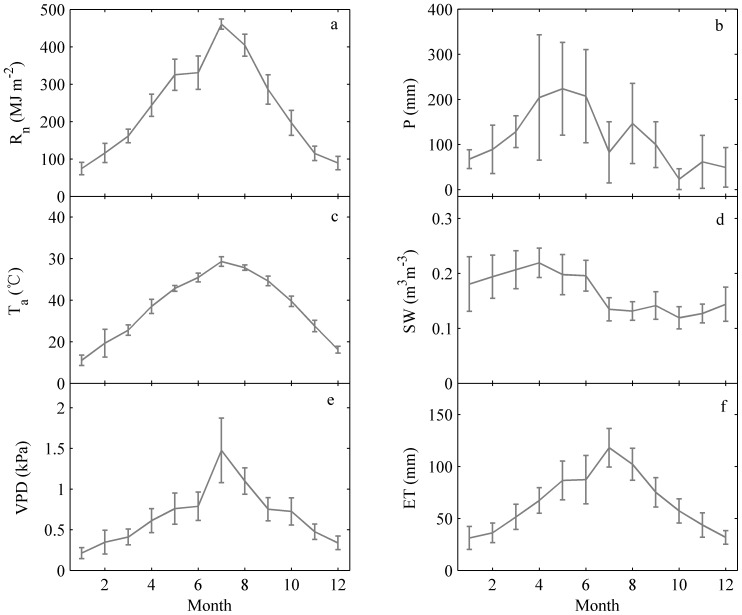
Multi-year (2003–2010) environmental conditions accumulated or averaged monthly: (a) net radiation (R_n_); (b) precipitation (P); (c) air temperature (T_a_); (d) soil water contents (SW) at 5 cm; (e) vapor pressure deficit (VPD) and (f) ET. The error bars represent the standard deviation.

The ET, net radiation (R_n_), air temperature (T_a_), vapor pressure deficit (VPD), precipitation (P) and soil water contents (SW) are characterized in Figure 3. The ecosystem ET and corresponding R_n_, T_a_, and VPD values showed a single peak, and reached their maximums in July (Fig. 3). The precipitation also had a strong secondary peak. The precipitation decreased in July as the air temperature reached a maximum. The asynchronous seasonality of temperature and precipitation, an important characteristic of the eastern Asian monsoon climate, often results in summer droughts in southern China. In addition, precipitation in the first half year was usually higher than in the second half of the year. The seasonal pattern in soil water content (SW) (at 5 cm) was not as obvious as the other factors. It remained between 0.12 m^3^ m^−3^ and 0.22 m^3^ m^−3^ and decreased after July, with values in the second half year generally lower than in the first half year, in accordance with precipitation pattern.

**Figure 4:**
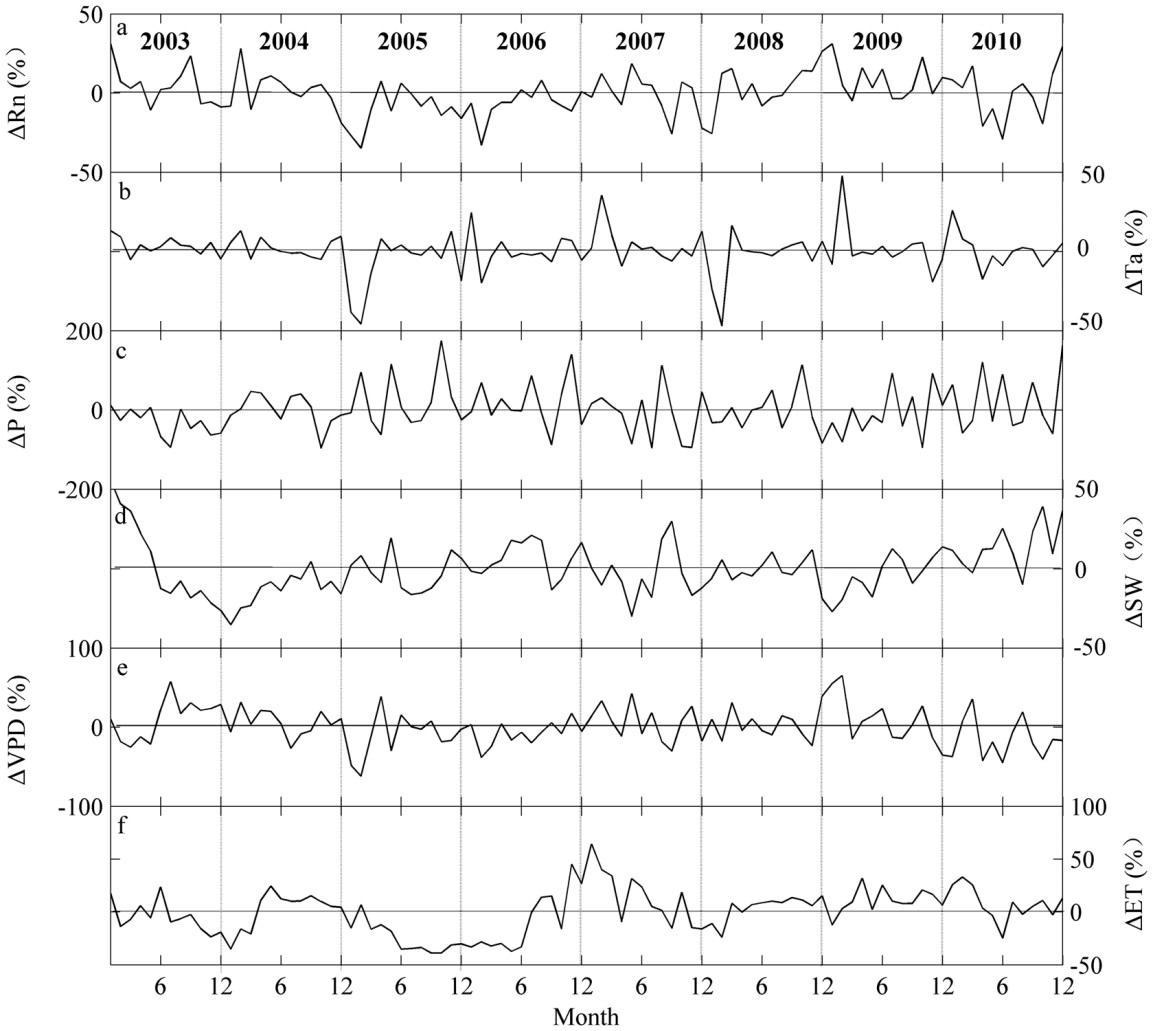
Monthly relative anomalies (%) of environmental factors and ET during 2003–2010: (a) net radiation (R_n_); (b) air temperature (T_a_); (c) precipitation (P); (d) soil water contents (SW) at 5 cm; (e) vapor pressure deficit (VPD) and (f) ET.

Figure 4 and Table 1 show the environmental condition and ET for each year at QYZ. Over the eight years, the average annual ET was 786.9±103.4 mm (CV = 13.1%) with a substantial variation, from 567.9 mm in 2005 to 876.5 mm in 2009. The years of 2007 and 2009 had the highest ET, whereas 2005 and 2006 had the lowest values (Table 1). Correspondingly, the T_a_ was higher in 2007 and 2009 in the early spring, and the R_n_ in 2009 was higher, with positive anomalies for ten months (Fig. 4). In 2004 and 2008, the climatic factors were close to the multi-year averages, except for a lower R_n_ and T_a_ in the early spring of 2008. In 2010, although the T_a_ was lower, the precipitation was abundant. In 2003, the negative anomalies in precipitation were large, and the asynchrony between precipitation and temperature resulted in a severe summer drought in June and July. In 2005 and 2006, the R_n_ was low in the first half year, and its annual total was approximately 6.4% lower in both years compared to multi-year average.

**Table 1:** Annual accumulated ET, R_n_, P and annual averaged T_a_ during 2003–2010.

	2003	2004	2005	2006	2007	2008	2009	2010	mean	CV
R_n_ (MJ m^−2^)	3036.0	2870.9	2620.8	2621.5	2777.5	2860.8	2939.1	2669.9	2799.6	5.5%
T_a_ (°C)	18.7	18.3	17.6	18.0	18.5	17.9	18.3	17.8	18.1	2.1%
P (mm)	944.9	1404.5	1455.4	1485.3	1318.7	1332.9	1253.6	1850.2	1380.7	18.4%
ET (mm)	759.3	837.0	567.9	718.5	866.4	832.60	876.6	837.0	786.9	13.1%

The last column presents coefficient of variation (CV).

The El Niño and La Niña events had significant impacts on the climatic factors. In the El Niño year (2003), the T_a_ was higher, but the precipitation was lower. In the La Niña year (2010), the precipitation was higher but the T_a_ was lower (Fig. 4b and c, Table 1). In addition, under the effects of El Niño events, the SW was lower in the second half year of 2003 and the first half of 2004, but the VPD was higher during this period. In contrast, under the influence of La Niña, the pattern in SW and VPD anomalies was opposite to that of the El Niño year (Fig. 4d and e). Correspondingly, in the El Niño year, the anomalies in the ET were negative for ten months, but in the La Niña year, the opposite trend was found, and the anomalies in the ET were positive for eleven months (Fig. 4f).

### Partitioning the sources of inter-annual variation of ET

To separate the sources of the inter-annual variability in ET, a statistical method (LUTs) was used to calculate the ET under different conditions. These approaches allowed the climatic drivers and ecosystem responses to change separately or together.

**Figure 5:**
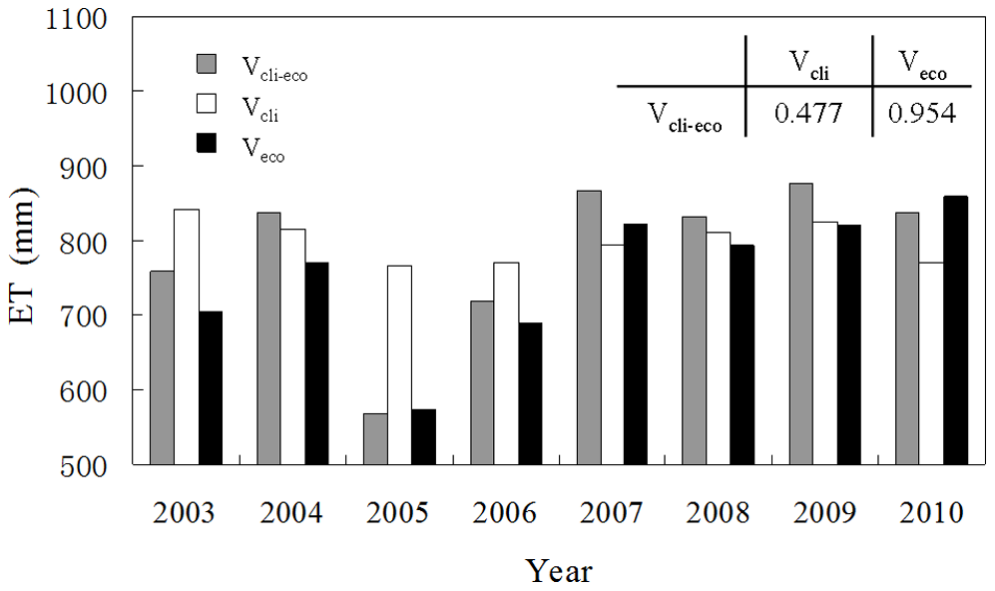
Yearly values of cumulative ET calculated through different approaches: V_cli-eco_ (for variable climate and variable ecosystem responses); V_cli_ (for variable climate); V_eco_ (for variable ecosystem responses). The correlation coefficients of the cumulative values calculated with the three approaches are also reported.

**Figure 6:**
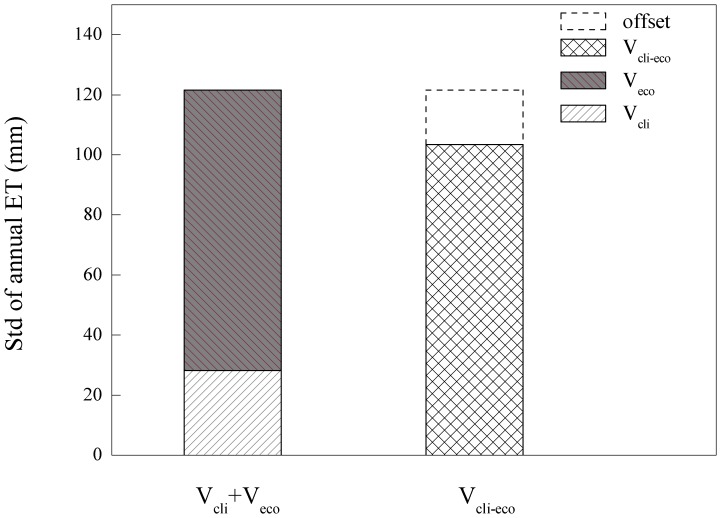
Standard deviation (Std) which represents inter-annual variability of annual cumulative ET calculated through different approaches: V_cli-eco_ (for variable climate and variable ecosystem responses); V_cli_ (for variable climate); V_eco_ (for variable ecosystem responses).

The annual ETs calculated through the three LUT approaches are presented in Figure 5. The average annual ETs were 798.5, 786.9, 754.1 mm for V_cli_, V_cli-eco_, V_eco_, respectively, and the corresponding inter-annual variabilities were 28.13, 103.40 and 93.50 mm (Fig. 6). V_cli_ showed the highest average annual ET and the lowest inter-annual variability. Compared to V_cli-eco_, which was almost equal to the measured ET, V_cli_ overestimated ET and dampened the inter-annual variability. This result implied that the ecosystem responses may offset climatic effects in the long term. In addition, the inter-annual variability of V_cli_ was only 27.2% of that produced by V_cli-eco_. At the same time, the correlation coefficient between the ETs of V_cli_ and V_cli-eco_ was only 0.48 (p = 0.2316), which indicated that the ecosystem had a noticeable resistance to direct changes in the external drivers. In contrast, V_eco_ underestimated ET and produced higher inter-annual variability. This indicated that the climatic factors drove the daily function of the ecosystems and maintained the normal ecosystem status, while the ecosystem responses furnished the main source of the inter-annual variability in ET.

Additionally, the inter-annual variability of ET was 28.13 mm as generated by variable climatic factors and was 93.50 mm as generated by ecosystem responses. If there were no interaction, the inter-annual variability of V_cli-eco_ should be 121.63 mm, but the combined value was 17.6% higher than the real result 103.40 mm (Fig. 6). Therefore, we can infer that the changes in ecosystem responses were counteracting the effects of climatic variations, an inference that agrees with the classical ecological theory.

## Discussion

### Direct and indirect effects of climatic factors on ET

Previous researchers have confirmed that variations in ET can be attributed to climatic factors and the biotic responses induced by environmental forcing [12], [15], [31], [61]. Although they are entangled and difficult to separate from each other, there are many studies that refer to the role biotic factors play in driving the variation in ET, especially in the long term [36], [62]. In this study, we used a statistical method to separate the effects of climatic drivers and ecosystem responses and quantified their contributions to the inter-annual variability of ET. The results underscore the prominent role that ecosystem responses play in the inter-annual variability of ET (Fig. 5 and Fig. 6). The ecosystem responses include many aspects, such as variation in LAI between years and variability in the photosynthetic ability of plants. These factors are sometimes difficult to measure and quantify over longer time scales. Fortunately, the indirect effects of climatic factors can, to some extent, reflect the ecosystem responses and help us understand the effects of ecosystem responses on the inter-annual variability of ET.

ET is composed of two components: evaporation and transpiration [63], [64]. When analyzing how biotic and abiotic factors affect ET, we should consider the effects on each part. Evaporation is mainly a physical process driven by radiation and VPD [65]. Transpiration is a biotic process, which may also be driven by environmental factors but that is mainly controlled by biological variables [66]. In this study, ET was found to be related to R_n_, T_a_, VPD, SW and precipitation, which is consistent with previous studies [13], [67]–[70]. The lag effects became apparent when the correlograms between annual ET and dominant climatic drivers were drawn. This phenomenon indicated that the ecosystem responses sometimes did not happen simultaneously with the environmental change.

Numerous connections between climatic factors and ET were observed in the short and long term [12]. On a shorter time scale, the direct effects of climatic factors are obvious. The correlation coefficients between climatic factors on monthly time scale were larger than they were on the annual scale (Table 2), which is similar to findings in studies of carbon fluxes [71], [72]. In addition, the ET showed a similar seasonal rhythm to the R_n_, T_a_ and VPD (Fig. 3). On the annual scale, the ET is still related to the climatic drivers. The annual ET was sometimes linked to the temperature and precipitation anomalies caused by the extreme climate events such as the El Niño event in 2003, which caused a low ET (27.6 mm lower than the average) (Fig. 4 and Table 1). However, generally the relationships between ET and the climatic factors were not as close as they were on shorter time scales. Moreover, when the statistical methods were employed to further analyze their relationship, important lag effects emerged.

**Table 2:** Correlation coefficients (R) between ET and some of the climatic drivers (R_n_, T_a_ and VPD) at monthly and annual scale (p represents the probability-value).

Factor	Monthly		Annual	
	R	p	R	p
R_n_	0.907	0.000	0.528	0.000
T_a_	0.858	0.000	0.261	0.016
VPD	0.791	0.000	0.209	0.055

**Figure 7:**
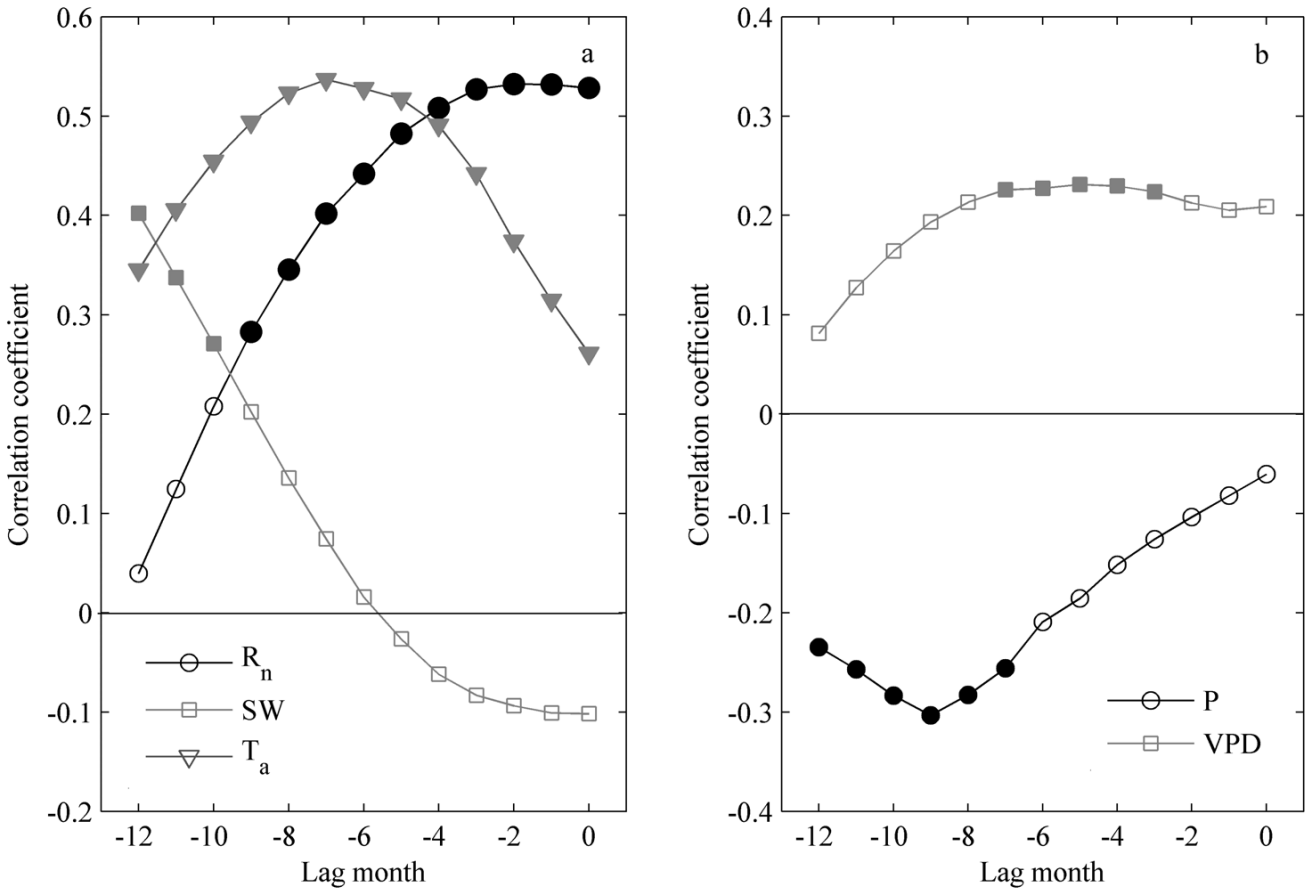
Correlation coefficients between annual ET and climatic drivers (R_n_, T_a_, P, SW and VPD). Correlograms were calculated by shifting the climatic driver backward one month at a time. The open signs represent that the coefficients are not significant while the closed signs mean significant (p<0.05).

Two mechanisms may explain the lag effects and lower correlation coefficients on longer time scales. First, the rhythm signals of finer time scales are disguised when the analysis is conducted on longer time scales. Second, the ecosystem requires time to respond to climatic factors and its modulating effect is prominent over longer period. This is what we refer to as ecosystem responses. However, the lag effects did not exist for all climatic factors (e.g., R_n_ and VPD, Fig. 7). The evaporation from the soil and the canopy surface respond quickly to changes of R_n_, as demonstrated by Monteith [27]. Meanwhile, R_n_ and VPD act on transpiration as a pulling force, and their effects were instantaneous [73].

As for T_a_, precipitation and SW, there are prominent lag effects as shown in Figure 7. This result agreed with some previous studies [44], and it can be observed as the indirect effect of climatic factors. The lag may be helpful in understanding the ecosystem responses. Here, we try to interpret the lag effects of T_a_, SW and precipitation. The relationship between ET and T_a_, showed a parabolic lag effect. The highest correlation coefficient showed up at 8 month lags, which is likely because the growth of forests often requires more time to respond to changes in temperature. This may be partly because of its impact on the growing season length (GSL) [74]. In this study area, the temperatures between April and November were generally above the threshold 5°C. Therefore, the GSL mainly depended on the temperature between December and March (Table 3). Zhang et al. [43] noted that lower temperatures in the early spring greatly reduce the annual net ecosystem exchange (NEE) of carbon by shortening the GSL in this study area. Because transpiration, which is the dominant component of ET, is coupled with photosynthesis, we tested the effect of GSL on ET. The resulting correlation coefficients between ET and GSL (T_a_≥5°C, in days) were as high as 0.56 (p = 0.1471, n = 8). This may partially explain the lower ET in 2005 and higher ET in 2007, because the GSL was shorter in 2005 and longer in 2007 (Table 3). This result is comparable to the results obtained by Zha et al. [36] which showed that the inter-annual variability of ET was controlled by early spring soil temperatures in three boreal forests and a grassland ecosystem.

**Table 3:** Air temperature (T_a_ °C) and growing season length (GSL, in days T_a_≥5°C) during 2003–2010.

Factor	Period	2003	2004	2005	2006	2007	2008	2009	2010
	Jan.	6.24	5.82	3.38	6.87	5.62	4.19	5.06	6.94
	Feb.	10.51	10.88	5.21	7.70	13.02	5.10	13.37	10.37
T_a_ (°C)	Mar.	12.10	12.13	11.00	12.35	14.08	14.79	12.48	13.30
	Apr.–Nov.	23.54	22.80	23.15	22.70	22.53	22.84	22.58	21.74
	Dec.	7.67	8.82	6.56	7.42	9.08	8.58	7.69	8.49
	Jan.	20	19	6	21	19	9	13	21
GSL	Feb.	23	22	11	20	28	14	26	19
(≥5°C)	Mar.	28	31	28	28	28	31	26	27
	Apr– Nov.	244	244	244	244	244	244	239	244
	Dec.	27	22	23	29	30	28	25	25

The highest correlation coefficients between ET and SW at 5 cm were found at time lags of 10–12 months. This indicated that the soil water conditions in a given year may have significant effects on ET in the following year. This phenomenon may be attributed to the rooting depth and available SW at deeper layers or potentially to plant responses that result in altered water use strategies under the soil water stress [25], [75], [76]. In our study site, the dominant tree species are Slash pine, Masson pine and Chinese fir. These pioneer species have deep root systems that may be capable of absorbing water from deeper layers, much like the plants in Florida scrub oak and pine flatwoods ecosystems that Bracho et al. [5] described as able to obtain water from deeper sources.

The correlation coefficients between precipitation and ET were negative on the annual scale. When the precipitation was high, forest ET was likely reduced because of the lower solar radiation due to frequent cloud cover. In dry years, as Lundquist and Loheide [22] described, the ecosystem typically has greater potential ET due to higher R_n_, higher T_a_, higher VPD and longer growing seasons. All of these factors provide strong driving forces for ET. In our study area, the precipitation is abundant on the annual scale. Even in dry years, the trees can access water in deeper layers, which may lead to the relatively high ET. Another possibility as suggested by Pejam et al. [30], is that larger precipitation events would contribute more to the surface run-off than to soil water storage. Because our study site is hilly, with red earth characterized by high clay soils with relatively poor infiltration rates, there may be high runoff when the rain is heavy [47].

### Contributions of seasonal variations to the inter-annual variation of ET

**Figure 8:**
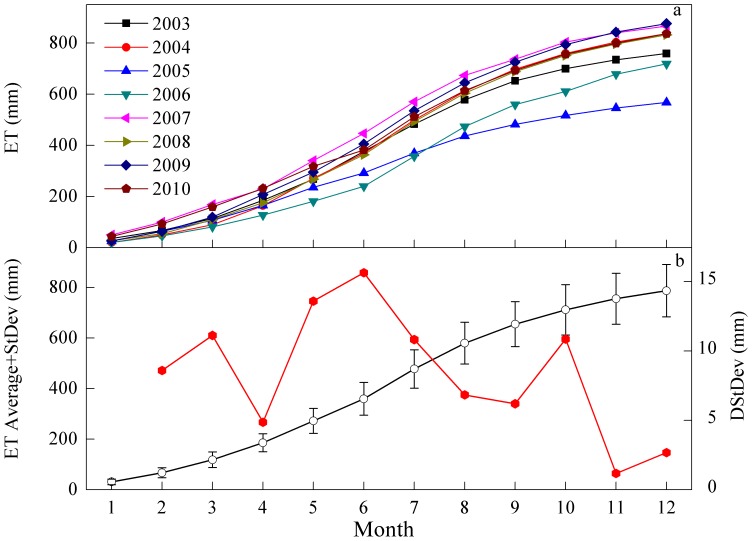
Cumulative ET for each year of 2003–2010 respectively (panel a); cumulative ET averaged over the observation periods and its standard deviation (black line) and monthly variations in standard deviation (red line and the axis on right) (panel b).

During the observed years, the ET of this ecosystem was positive throughout the year (Fig. 8a). Although the ET for all eight years had a similar cumulative trend, large inter-annual variability emerged when comparing the cumulative curves of different years (Fig. 8a). The inter-annual variability of ET in this subtropical coniferous plantation was also relatively large when compared to the other ecosystems (Table 4).

**Table 4:** Coefficient of variation (CV) of ET at flux sites with over 3 years' observations.

Forest type	Location	Mean annual ET (mm)	CV (%)	Observation period	Source
Boreal deciduous coniferous	62°15′18″N, 129°14′29″E	196.1	10	1998–2006	[37]
Boreal broadleaved deciduous	53°37′48″N, 106°11′24″W	405	21	1998–2006	[36]
Boreal coniferous	53°59′24″N, 105°7′12″W	374	9	1998–2006	[36]
	53°55′12″N, 104°41′24″E	300	7	1998–2006	
Temperate broadleaved deciduous	36°58′41″N, 79°05′39″W	633	4.1	2002–2005	[41]
Temperate broadleaved deciduous	35°57′30″N, 84°17′15″W	567.2	13	1995–1997	[31]
Temperate coniferous	34°58′N, 136°00′E	729	4.4	2001–2003	[39]
Subtropical coniferous	26°44′29″N, 115°03′29″E	787	13.1	2003–2010	This study
Tropical rainforest	21°55′39″N, 101°15′55″E	1029	2.8	2003–2006	[20]
	4°20′N, 113°50′E	1323	5.6	2000–2009	

At the beginning of each year, from January to mid-March, the ET increased slowly at less than 2 mm d^−1^. From mid-March to mid-July, the cumulative ET rose sharply, up to 4 mm d^−1^ in June, and then gradually declined through the end of the year. With respect to the increasing rate of ET, the absolute difference in ET among years was not large in the beginning of the year. Since the growing season, accompanied with the intensive transpiration, the discrepancies in cumulative curves became larger (Fig. 8a). However, the increases in rates could not fully account for the contributions to the inter-annual variability in the ET. Therefore, to evaluate the contributions of different phenological phases to the variability in annual ET, the difference between the standard deviation of cumulative ET from a given month and its preceding one (DstDev) was calculated to quantify the contribution of each month to the observed inter-annual variability of the ET. Over the whole year, the DstDev had three peaks, in March, May–June and October (Fig. 8b), which coincide with the seasonal transitions.

In subtropical China, March is the beginning of the spring, and new twigs generally sprout as the plant leaves expand. Accompanying this phenological transition, the cumulative ET increased rapidly (Fig. 8a) because of the great increase in transpiration [77]. However, in accordance with variations of early spring temperatures [43], the phenological calendars varied among years. For example, in 2005 the low temperature in spring postponed the twig and leaf emergence and caused phase lags in the enhanced vegetation index (EVI) time series [43]. Therefore, the phenological transition and the advances or lags in growing seasons gave the ET in March a bigger role in the inter-annual variability of ET. Zha et al. [36] found a similar effect in the Siberian forests, years with warmer spring temperatures had a greater overall ET because of earlier leaf emergence. Hollinger et al. [78] also found a similar phenomenon in a carbon flux study in forests. They noted that the climatic conditions in the spring largely influenced the inter-annual variability of NEE. The ecosystem they studied absorbed more atmospheric carbon dioxide in years with warm spring and less carbon dioxide in years with cold spring.

The variations of ET in May and June had the largest contribution to the inter-annual variability of ET in this study. This may have occurred for the following reasons. First, there was substantial variability in the ecosystem structure and function in this period. The ecosystem behaved similarly in this period every year, but the differences were great between years. For example, the LAI or EVI would level off during this period each year, but when compared between years, large differences emerged [43]. Transpiration, which accounts for 70–80% of ET [15], [79], became more active in this period [77]. Therefore, under the coactions of these two factors, the ET varied greatly in this period between years. Second, this period marks the transition between wet and dry seasons, which occurs with the seasonal reversal of the monsoon circulation features [80]. The variability in the onset of the monsoon [81], [82] led to a high inter-annual variability in the climatic factors. The climatic factors and variable ecosystem responses combined to make this period the most influential on the inter-annual variability of ET.

In October, the DstDev increased abruptly, which might have been caused by the inter-annual variability in senescence time of old needles and the retreat time of the monsoon [81]. After October, the lower Dstdev may have been driven by the lowering activity of the plants. Therefore, the underlying mechanisms that make these seasonal transitions important to the inter-annual variability of ET may be the coactions of climatic factors and ecosystem responses. This is consistent with the view that seasonal transitions have great influence on fluxes and energy exchange and that they play an important role in inter-annual variability [83], [84].

### The role of ecosystem responses in long term studies

Previous studies have generally focused more on the effects of climatic factors. Although some researchers have considered the biotic factors such as canopy conductance to be as important as climatic factors [31], [85], [86], the biotic effects on ET have not generally received adequate consideration [12]. In our study, we interpreted all biotic factors as an integrated part of ecosystem responses, and we separated the sources of the inter-annual variability of ET into those that were induced by climate and those produced by the ecosystem responses. The results suggested that ecosystem responses were largely responsible for the inter-annual variability of ET (Fig. 5 and Fig. 6). As discussed above, both of the indirect effects of the climatic factors and the three peaks in variability contributed to the inter-annual variability of ET as shown in Figure 8, underscore the importance of ecosystem responses. By taking ecosystem responses into consideration, more accurate estimations of ET may be made, particularly in years where climatic drivers seem unable to capture the variation in ET (Fig. 5). Our results indicate that the ecosystem ET may respond directly to the climatic forces, but it may also provide some buffering effects because the ecosystems requires time to respond to external stimulations and adjust its functions and structures [87].

Currently, several types of models are used to estimate and predict ET worldwide [12]. However, most of those models tend to underestimate its inter-annual variability [88]. Komatsu et al. [40] suggested that the models can be roughly classified into two groups: simple models that use only climatic factors as input variables [89] and complex models that employ more process-based formulations including the Penman–Monteith equation or the bulk equation [15]. Although the process-based models give limited consideration to biotic factors, they are still limited in their ability to estimate the inter-annual variability of ET. Our results indicate that the ecosystem responses are more important to the inter-annual variability of ET than the environmental drivers, which implies that to more precisely evaluate ecosystem ET, the features of ecosystem responses should be considered alongside the climatic factors, especially for longer time scales.

There is also potential for an effect of CO_2_ fertilization on the inter-annual variation of ET under climate change context. It has been reported that the elevated CO_2_ concentrations may enhance photosynthesis in forests [90]. They may also affect the transpiration, the dominant component of ET, because photosynthesis and transpiration are coupled in the stomata [12]. In addition, elevated CO_2_ concentration may affect the growing season length [91]–[92] and induce changes in the ecosystem functions [93]–[94]. These changes in ecosystem responses may in turn increase the inter-annual variability of ET. Recently, the atmospheric CO_2_ concentration has been increasing by approximately 2 ppm per year [95], and it rose approximately 14 ppm during our study period (2003–2010) [96]. Compared to CO_2_ enrichment experiments and models, this increase is minor, and therefore the effect of CO_2_ fertilization on ET might not have been captured by our study. However, in the context of climate change, the rising atmospheric CO_2_ concentrations may affect ET in the long term by affecting plants and by magnifying ecosystem responses (e.g., LAI, GSL) [91]. Such scenarios should be discussed in the future.

However, ecosystem responses are complicated and difficult to quantify. To demonstrate the underlying mechanisms, accurate knowledge of how the components of ET (e.g., soil evaporation, canopy evaporation and transpiration) respond to climatic drivers is vital and should be targeted for future investigations of ET. These efforts will require more comprehensive models and additional data. Though some processes respond quickly to variation in climatic factors, other biogeochemical processes may need a longer time to respond to the environmental changes [72]. Thus, long-term physiological and biogeochemical research is needed to elucidate the bioprocesses and the underlying mechanisms.

## Conclusions

1. The ET of a coniferous plantation in subtropical China showed a strong seasonal pattern, consistent with the single peak of climatic factors such as R_n_, T_a_, VPD,which reached their maximums in July. During the observation period from 2003 to 2010, the mean value of annual ET was 786.9 mm. A relatively large inter-annual variability with a standard deviation of 103.40 mm (CV = 13.1%) was detected.
2. The main source of the inter-annual variability of ET was ecosystem responses, according to the LUT results. The inter-annual variability of ET caused by climate variability and that caused by changes in ecosystem responses had a negative interaction.
3. On the annual scale, the main meteorological drivers for ET were R_n_ and T_a_. The R_n_ affected ET directly without a lag effect. In contrast, the correlation coefficients between ET and T_a_, SW and precipitation showed significant lag effects which reflect the ecosystem responses.
4. The variation during seasonal transitions in March, May–June, and October contributed heavily to the inter-annual variability of ET, which could be attributed to the coactions of climatic factors and ecosystem responses.
